# Supplementary material for: PNSA, a Novel C-Terminal Inhibitor of HSP90, Reverses Epithelial–Mesenchymal Transition and Suppresses Metastasis of Breast Cancer Cells In Vitro
Source: Mar Drugs. 2021 Feb 20;19(2):117. doi: 10.3390/md19020117 (PMC7923764; doi:10.3390/md19020117)
Supplement: Supplementary file 1 [file marinedrugs-19-00117-s001.pdf]

PNSA, a novel C-terminal inhibitor of HSP90, reverses epithelial-mesenchymal transition and suppresses metastasis of breast cancer cells in vitro

Aotong Zhang[1], Xin Qi[1], Fu Du[1], Guojian Zhang[1,2], Dehai Li[1,2] and Jing Li[1,2]

[1] Key Laboratory of Marine Drugs, Chinese Ministry of Education, School of Medicine and Pharmacy, Ocean University of China, Qingdao 266003, China

[2] Open Studio for Druggability Research of Marine Natural Products, Laboratory for Marine Drugs and Bioproducts, Qingdao National Laboratory for Marine Science and Technology, Qingdao 266237, China

* Correspondence: authors: Key Laboratory of Marine Drugs, Chinese Ministry of Education, School of Medicine and Pharmacy, Ocean University of China, Qingdao 266003, China. lijing_ouc@ouc.edu.cn (Jing.Li); Tel: +81-0532-82031980; Fax: +81-0532-82031980;

Supplemental Information Table of Contents

The structure elucidation of PNSA

Figure S1: HPLC-UV analysis of PNSA.

Table S1: Peak purity report of PNSA.

Table S2: HPLC gradient profile for detection of PNSA.

Figure S2: The ^{1}H NMR (500 MHz) spectrum of PNSA in CD_3OD.

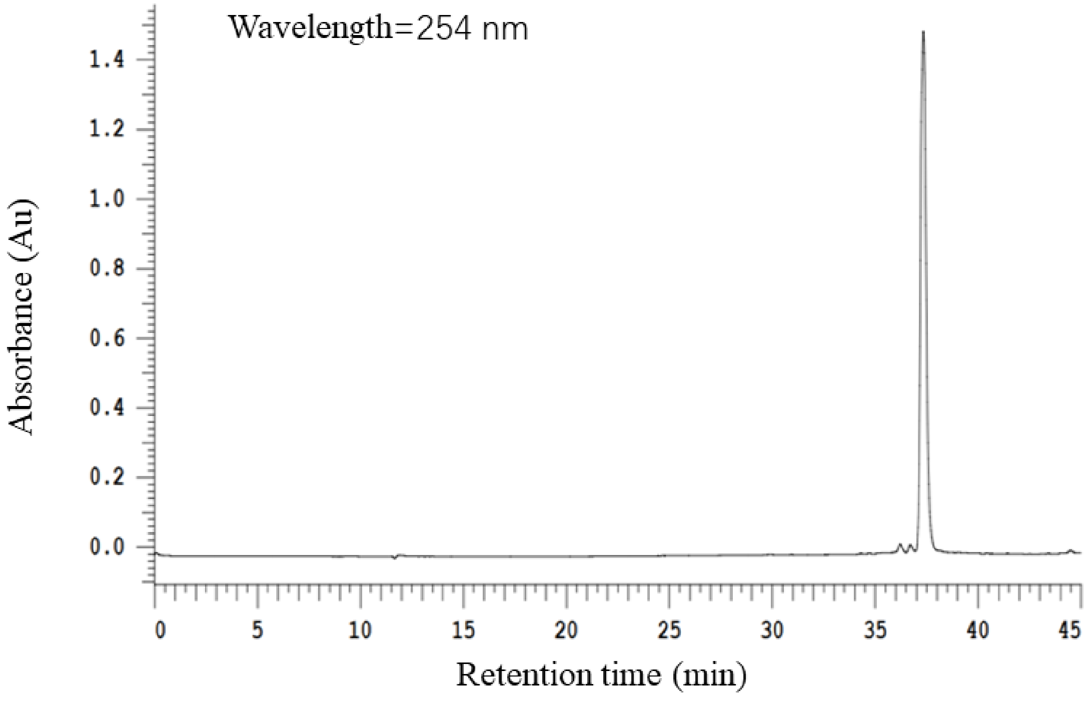

Supplementary Figure S1. HPLC-UV analysis of PNSA. The PNSA was analyzed by HPLC made by the HITACHI company equipped with a 5430 diode array detector and a

C18 column (YMC-Pack ODS-A, 4.6 × 250 mm, 5 μm, 1 mL/min) by using stepwise gradient elution with 5–100% MeOH–H_2O (0-5 min: 5%; 5-35 min: 5%-100%; 35-40 min: 100%; 40.1-45 min 5%).

Supplementary Table S1. Peak purity report of PNSA.

No.	RT	Area	Concentration
1	36.200	148084	1.127
2	37.333	12988293	98.873
		13136377	100.00

Supplementary Table S2. HPLC gradient profile for detection of PNSA (A: Methanol B: Water).

Time (min)	Solvent A (%)	Solvent B (%)	Flow (mL/min)
0.0	5.0	95.0	1.000
5.0	5.0	95.0	1.000
35.0	100.0	0.0	1.000
40.0	100.0	0.0	1.000
40.1	5.0	95.0	1.000
45.0	5.0	95.0	1.000

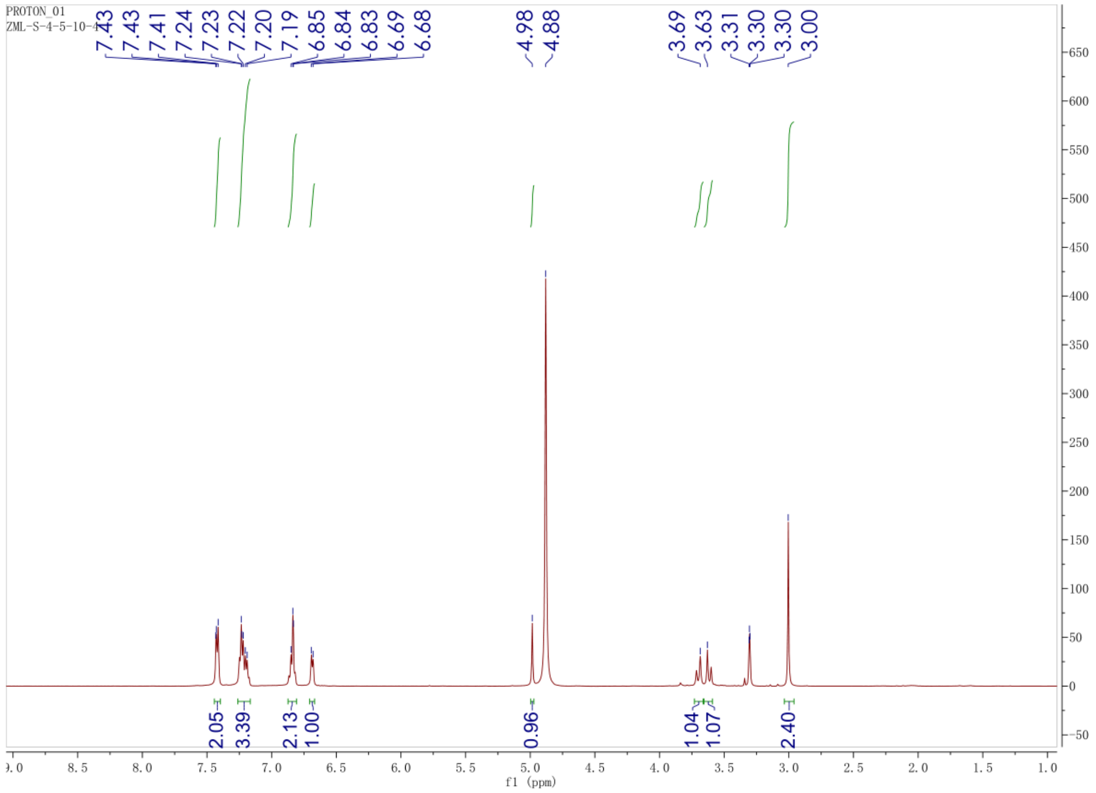

Supplementary Figure S2. The ^1H NMR (500 MHz) spectrum of PNSA in CD_3OD.
